# Mucosal snare resection-endoscopic submucosal excavation: a novel technology

**DOI:** 10.1055/a-2261-7532

**Published:** 2024-02-22

**Authors:** Yongli Ye, Yiping Hong, Wei Wei, Xiaowei Ji, Xinliang Lu

**Affiliations:** 1 Endoscopy Center, The Second Affiliated Hospital, Zhejiang University School of Medicine, Hangzhou, China; 2 Department of Gastroenterology, The Affiliated Jinhua Hospital, Zhejiang University School of Medicine, Jinhua, China; 3 Department of Gastroenterology, The Second Affiliated Hospital, Zhejiang University School of Medicine, Hangzhou, China

Endoscopic submucosal excavation (ESE) is a less invasive therapeutic alternative to surgical resection for the removal of gastric submucosal tumors 1 . The conventional procedural steps of this technique all involve cautery markings to delineate the target lesion, followed by submucosal injection and the resection of the lesion using a hook knife, insulation-tipped knife, or dual knife, and finally, closure of the mucosal incision using clips 2 . We report a novel variant ESE technique, mucosal snare resection-endoscopic submucosal excavation (MSR-ESE), for a lower technical burden and shorter procedure time compared with ESE.

MSR-ESE was successfully carried out as follows. First, the surface mucosa of the lesion was enclosed by the snare and electrocoagulation excision was performed without prior submucosal injection ( Fig. 1 ). With gradual exposure, the lesion was completely dissected along its edge and above the muscularis propria by an insulation-tipped knife ( Fig. 2 ). After complete resection and retrieval of the lesion, the mucosal incision was closed tightly by metal clips ( Fig. 3 ). The total operation time was 18 minutes, and no bleeding or perforation complications occurred ( Fig. 4 , Video 1 ).

**Fig. 1:**
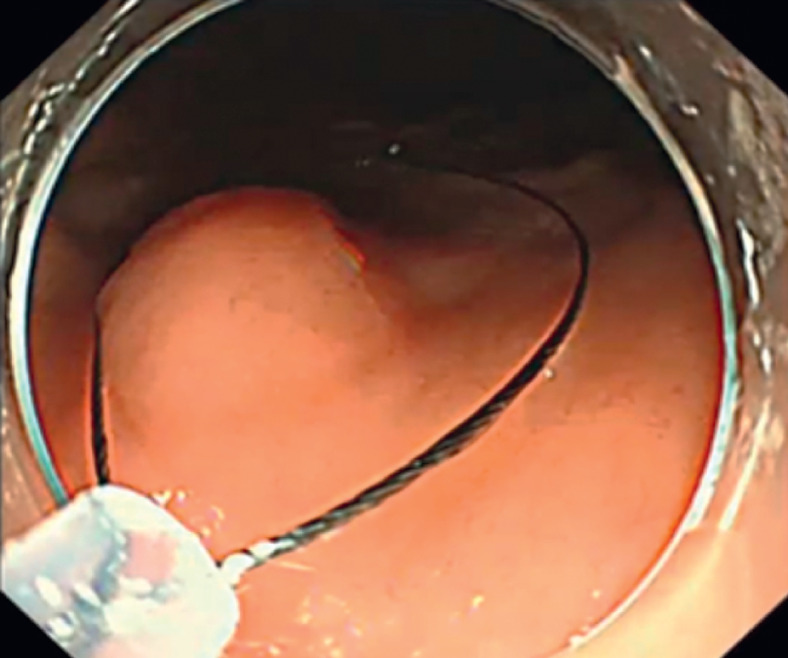
The lesion was enclosed by the snare without submucosal injection.

**Fig. 2:**
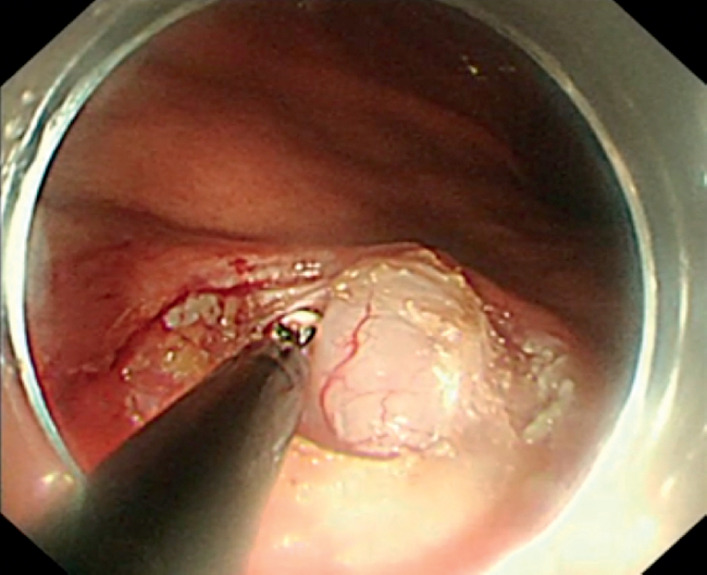
The lesion was dissected using an insulation-tipped knife.

**Fig. 3:**
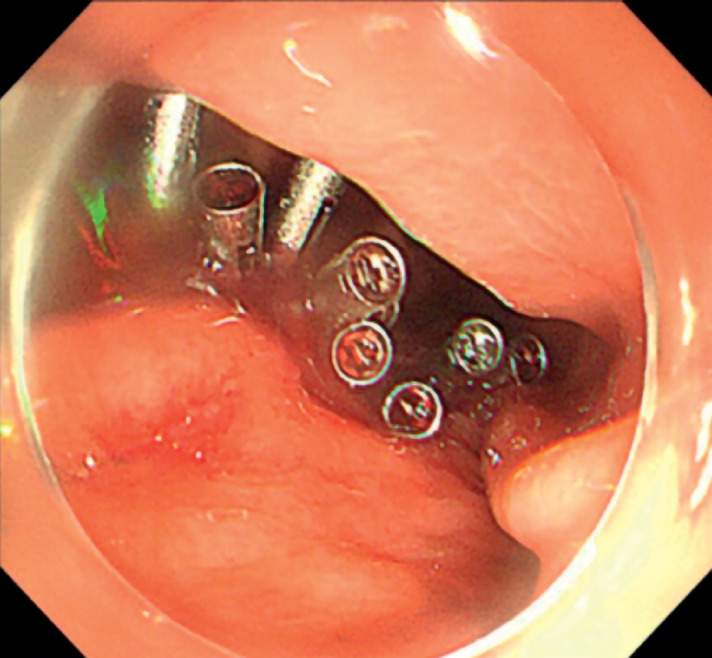
The clips tightly clamped the mucosa for wound closure.

**Fig. 4:**
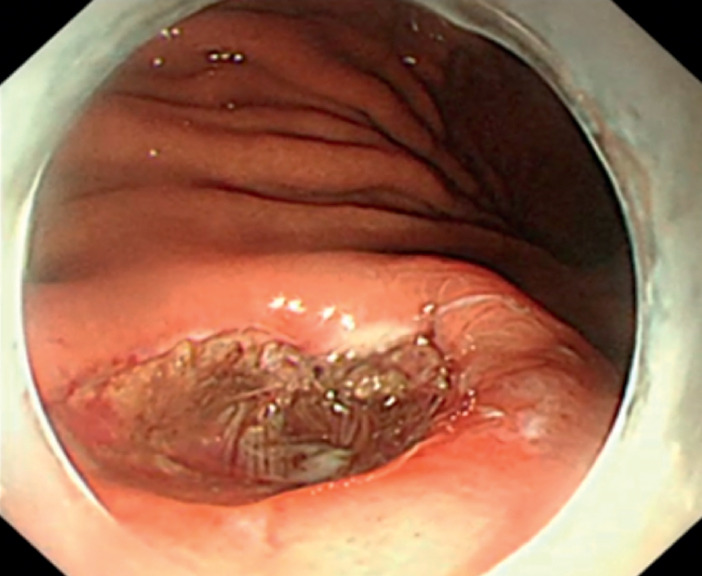
A submucosal tumor was successfully resected using mucosal snare resection-endoscopic submucosal excavation.

Mucosal snare resection-endoscopic submucosal excavation.Video 1

MSR-ESE eliminates the need for electrocoagulation marking and submucosal injection. Our department has performed more than 10 cases successfully. Thus, in the future, MSR-ESE may be considered a safe, time-saving, and effective option for submucosal tumors.

Endoscopy_UCTN_Code_TTT_1AQ_2AC

## Notes

Endoscopy E-Videos https://eref.thieme.de/e-videos
*E-Videos* is an open access online section of the journal *Endoscopy* , reporting on interesting cases and new techniques in gastroenterological endoscopy. All papers include a high-quality video and are published with a Creative Commons CC-BY license. Endoscopy E-Videos qualify for HINARI discounts and waivers and eligibility is automatically checked during the submission process. We grant 100% waivers to articles whose corresponding authors are based in Group A countries and 50% waivers to those who are based in Group B countries as classified by Research4Life (see: https://www.research4life.org/access/eligibility/ ). This section has its own submission website at https://mc.manuscriptcentral.com/e-videos .

## References

[1] Li B Chen T Qi ZP Efficacy and safety of endoscopic resection for small submucosal tumors originating from the muscularis propria layer in the gastric fundus Surg Endosc 2019 33 2553 2561 30478693 10.1007/s00464-018-6549-6

[2] Cheng BQ Du C Li HK Endoscopic resection of gastrointestinal stromal tumors J Dig Dis 2023 10.1111/1751-2980.13217 37584643

